# 1,3-Bis(4-bromo­phen­yl)-1*H*-imidazol-3-ium tetra­fluoro­borate

**DOI:** 10.1107/S2414314626002531

**Published:** 2026-03-17

**Authors:** Halliru Ibrahim, Sizwe J. Zamisa, Muhammad D. Bala, Pinkie Ntola

**Affiliations:** a Department of Chemistry, Durban University of Technology, PO Box 1334, Durban, 4000, South Africa; b School of Agriculture and Science, Discipline of Chemistry, University of KwaZulu-Natal, Private Bag X54001, Westville, Durban, 4000, South Africa

**Keywords:** crystal structure, halogen bonds, imidazolium, tetra­fluoro­borate salt

## Abstract

The crystal of the title salt, C_15_H_11_Br_2_N_2_^+^·BF_4_^−^, contains one half of a 1,3-bis­(4-bromo­phen­yl)imidazolium cation and one half of a tetra­fluoro­borate anion in the asymmetric unit; the complete ions are generated by crystallographic twofold symmetry. The imidazolium cation adopts a *syn*-periplanar conformation, with the 4-bromo­phenyl rings inclined to the central imidazolium ring by 36.04 (4)°. In the crystal, the tetra­fluoro­borate anion participates in structure-directing halogen-bonding contacts through F⋯Br and F⋯π(phen­yl) inter­actions. These are complemented by inter­molecular π–π and Br⋯π inter­actions, which assemble the ions into two-dimensional supra­molecular sheets lying parallel to the *ac* plane. The sheets are further linked by C—H⋯F hydrogen bonds, forming ring motifs of graph sets *R*^2^_2_(7) and *R*^2^_1_(4) within a three-dimensional supra­molecular network.

## Abstract 2

The crystal structure of the title imidazolium salt exhibits structure-directing, inter­molecular halogen-bonding patterns through F⋯Br and F⋯π_phen­yl_ inter­actions *via* the tetra­fluoro­borate anion.

## Graphical abstract

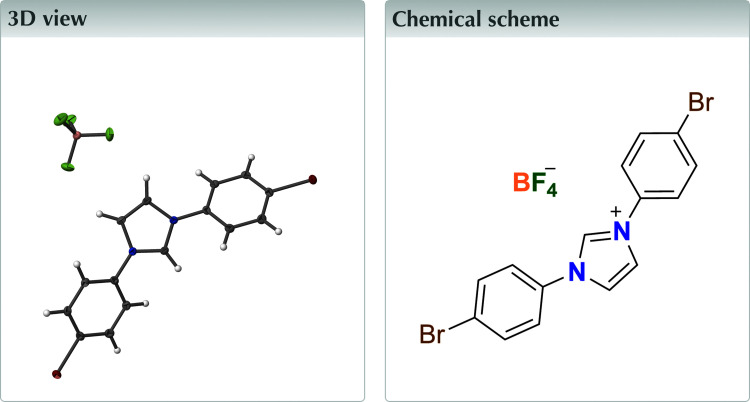

## Structure description

The title compound is a 1,3-di­aryl­substituted imidazolium salt, which was originally synthesized *via* a method involving mechano-grinding and acidification (Ikhile *et al.*, 2011 ▸). The synthetic method employed herein is a ‘green’ modification of the initial method (Arduengo *et al.*, 1992 ▸). While the synthesis of the title compound has been reported (Ikhile *et al.*, 2011 ▸), crystallographic details and those of analogous 1,3-bis­(4-halophen­yl)imidazolium tetra­fluoro­borates are not available. The title compound and its related analogues bearing ferrocenyl moieties have found application as green catalysts for the transfer hydrogenation of ketones (Ikhile *et al.*, 2012 ▸, 2013 ▸). Recent work on similar 1,3-di­aryl­imidazoliumm salts (Ndlovu *et al.*, 2017 ▸) and reviews on the biological activity of non-heteroatom functionalized azolium salts (Patil *et al.*, 2020 ▸; Fletcher *et al.*, 2018 ▸; Mercs & Albrecht, 2010 ▸) have also provided evidence on the structure/activity trends in their well established potential as anti-fungal, anti-bacterial and anti-proliferative agents. As part of our work in developing new imidazolium derivatives with impressive anti-microbial activities (Kadafour *et al.*, 2022 ▸; Ndlovu *et al.*, 2017 ▸), we synthesized the title compound and determined its crystal structure.

**Figure 1:**
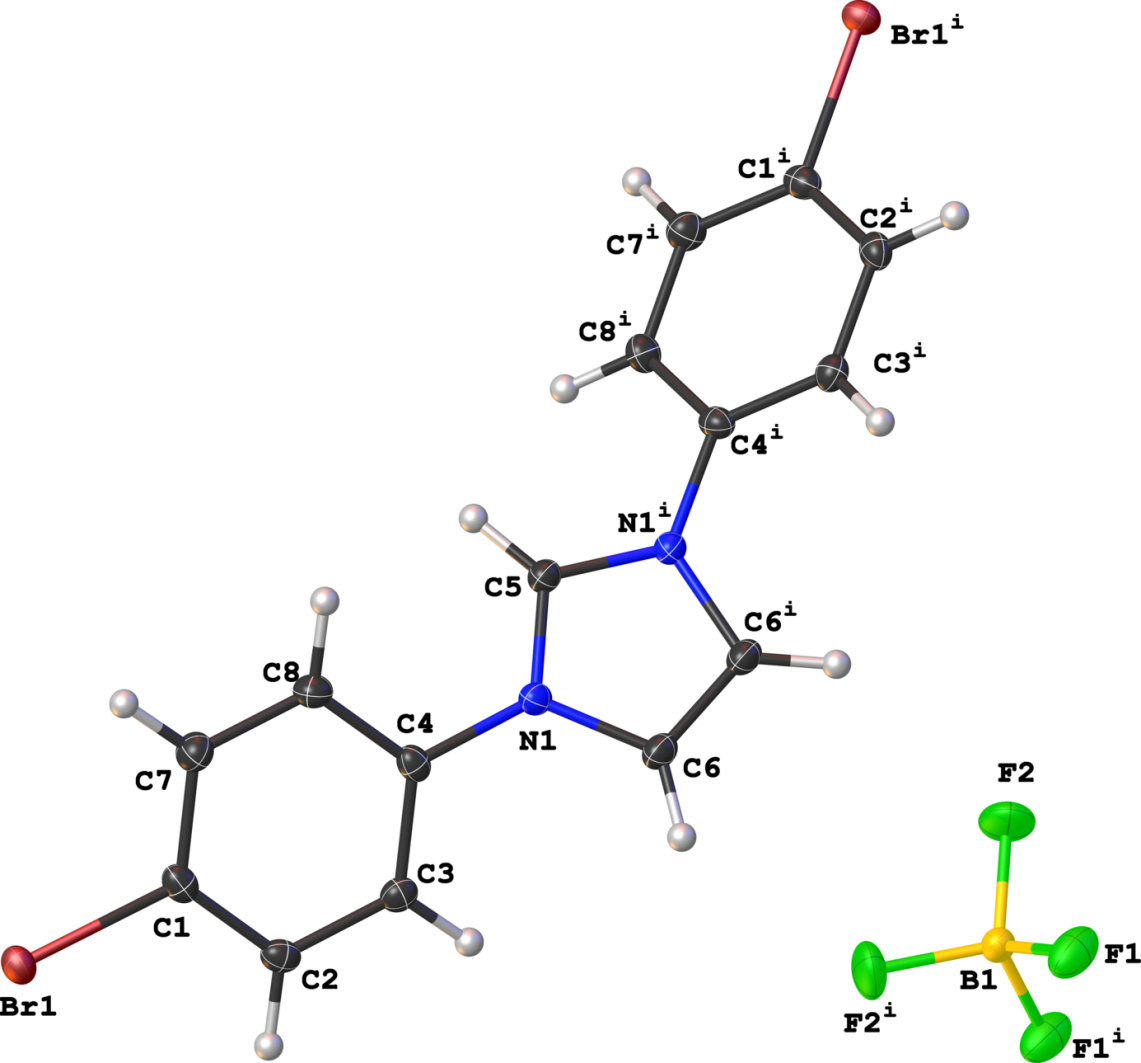
Mol­ecular structure of the title compound showing the atom-numbering scheme and displacement ellipsoids drawn at the 50% probability level. Symmetry code: (i) −*x* + 1, *y*, −*z* + 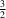.

The asymmetric unit of the title compound comprises half a cationic 1,3-bis­(4-bromo­phen­yl)imidazolium species and half a tetra­fluoro­borate counter-ion, with the complete ions being generated by a *C*_2_ rotation axis that runs parallel to the C5—H5 bond (Fig. 1 ▸). The cationic species adopts a *syn*-periplanar conformation with the dihedral angle between the mean planes of the central imidazolium ring and the 4-bromo­phenyl wingtip being 36.04 (4)°, which is wider than that observed in the hydrated chloride analogue of the title compound, *i.e*. 2.9 (1)° (Garden *et al.*, 2010 ▸).

**Figure 2:**
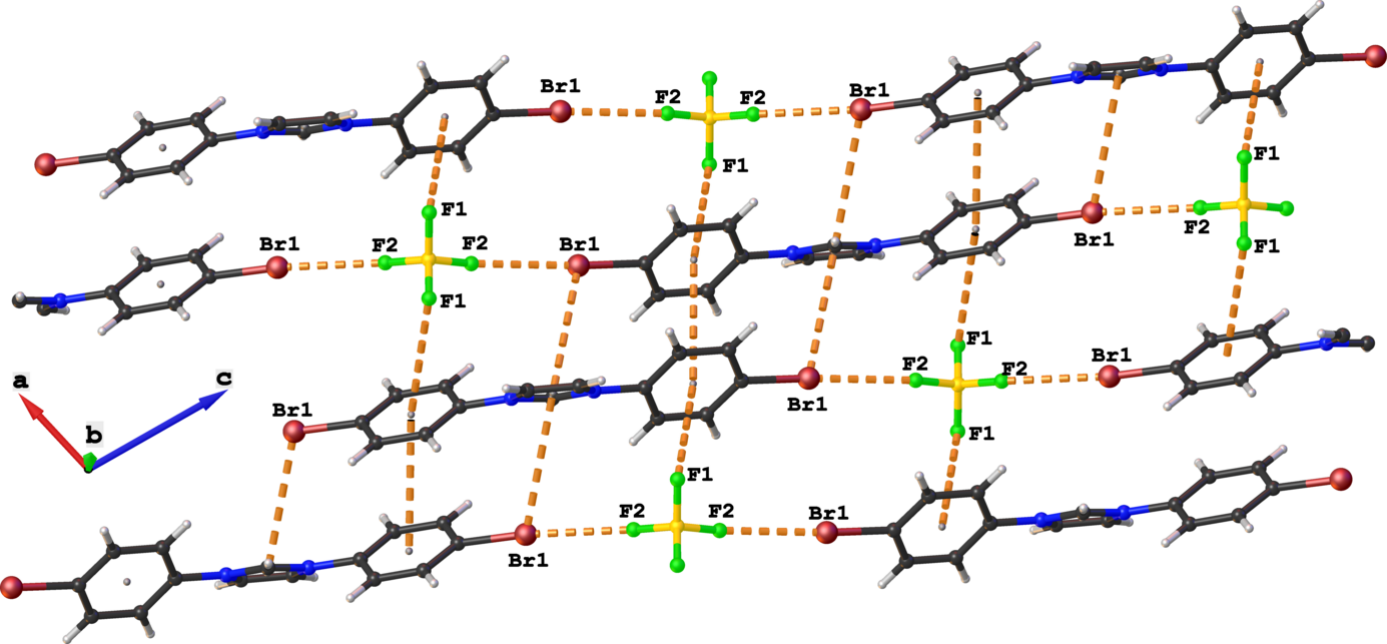
Representation of π(phen­yl)–π(phen­yl), π(phen­yl)⋯Br and F⋯Br inter­actions in the crystal of the title compound.

**Figure 3:**
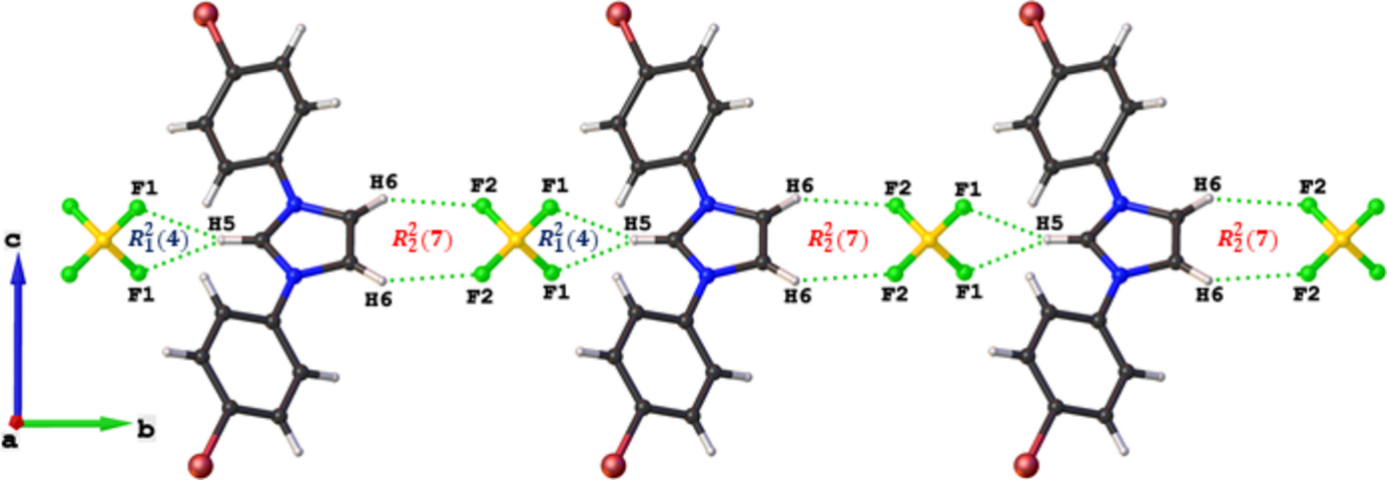
Representation of C—H⋯F hydrogen bonds in the crystal of the title compound.

The extended structure features halogen bonding that is driven by the tetra­fluoro­borate moiety *via* F1⋯π(phen­yl) [F1⋯*Cg*(phen­yl) = 3.5669 (12) Å, symmetry operation: 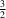 − *x*, 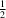 + *y*, 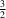 − *z* and F2⋯Br1 (F2⋯Br1 = 2.8890 (12) Å, symmetry code: 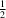 + *x*, 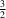 − *y*, −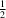 + *z*] inter­actions. In conjunction with the halogen-bonding patterns involving F atoms, inter­molecular π(phen­yl)–π(phen­yl) [*Cg*⋯*Cg* = 3.6720 (9) Å, symmetry operation: 1 − *x*, 1 − *y*, 1 − *z*] and π(phen­yl)⋯Br1 [Br⋯π = 3.9061 (6) Å, symmetry operation: 1 − *x*, 1 − *y*, 1-*z; x*, 1 − *y*, −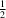 − *z*) inter­actions occur within two-dimensional supra­molecular networks parallel to the *ac* plane (Fig. 2 ▸). Finally, inter­molecular C—H⋯F hydrogen bonds with graph-set descriptors 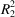
(7) and 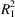
(4), link the two-dimensional supra­molecular networks along the *b* axis within a three-dimensional framework (Table 1 ▸, Fig. 3 ▸).

## Synthesis and crystallization

The details on the synthesis of the title compound have been reported (Ikhile *et al.*, 2011 ▸). The starting materials were 1,3-bis­(4-bromo­phen­yl)ethyl­enedi­imine (1.5 g; 4.10 mmol), paraformaldehyde (0.123 g: 4.10 mmol) and HBF_4_ (0.54 ml; 0.36 g; 4.10 mmol). Brown precipitate: 0.9603 g (yield: 62%); m.p. 181 °C; IR (ATR cm^−1^): 3126, 1590, 1491, 1401, 1295, 1122, 1059, 1015, 988, 960, 827, 617, 592, 523. ^1^H NMR (400 MHz, DMSO-*d*_6_): δ 7.88 (4H, *d*, *J* = 8.9, Ar—*H*), 7.97 (4H, *d*, *J* = 8.9, Ar—*H*), 8.57 (2H, *s*, Imid-C*H*=C*H*) and 10.37 p.p.m. (1H, *s*, NC*H*N). All other spectroscopic data matched those previously reported. Suitable crystals for X-ray diffraction analysis were grown by layering the DMSO solution with diethyl ether.

## Refinement

Crystal data, data collection and structure refinement details are summarized in Table 2 ▸.

## Supplementary Material

Crystal structure: contains datablock(s) I. DOI: 10.1107/S2414314626002531/tk4122sup1.cif

Structure factors: contains datablock(s) I. DOI: 10.1107/S2414314626002531/tk4122Isup2.hkl

Supporting information file. DOI: 10.1107/S2414314626002531/tk4122Isup3.cml

CCDC reference: 2536403

Additional supporting information:  crystallographic information; 3D view; checkCIF report

## Figures and Tables

**Table 1:** Hydrogen-bond geometry (Å, °)

*D*—H⋯*A*	*D*—H	H⋯*A*	*D*⋯*A*	*D*—H⋯*A*
C5—H5⋯F1^i^	0.95	2.29	3.156 (2)	151
C6—H6⋯F2^ii^	0.95	2.39	3.1854 (19)	140

Symmetry codes: (i) 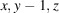; (ii) 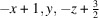.

**Table 2:** Experimental details

Crystal data	
Chemical formula	C_15_H_11_Br_2_N_2_^+^·BF_4_^−^
*M*_r_	465.89
Crystal system, space group	Monoclinic, *C*2/*c*
Temperature (K)	100
*a*, *b*, *c* (Å)	9.5407 (2), 9.8272 (2), 17.0451 (3)
β (°)	102.878 (1)
*V* (Å^3^)	1557.92 (5)
*Z*	4
Radiation type	Mo *K*α
μ (mm^−1^)	5.25
Crystal size (mm)	0.37 × 0.28 × 0.21

Data collection	
Diffractometer	Bruker SMART APEXII area detector
Absorption correction	Multi-scan (*SADABS*; Krause *et al.*, 2015 ▸)
*T*_min_, *T*_max_	0.618, 0.746
No. of measured, independent and observed [*I* > 2σ(*I*)] reflections	9293, 1922, 1748
*R*_int_	0.023
(sin θ/λ)_max_ (Å^−1^)	0.667

Refinement	
*R*[*F*^2^ > 2σ(*F*^2^)], *wR*(*F*^2^), *S*	0.017, 0.038, 1.06
No. of reflections	1922
No. of parameters	110
H-atom treatment	H-atom parameters constrained
Δρ_max_, Δρ_min_ (e Å^−3^)	0.37, −0.29

Computer programs: *APEX2*, *COSMO* and *SAINT* (Bruker, 2009 ▸), *SHELXS2013* (Sheldrick, 2008 ▸), *SHELXL2018/3* (Sheldrick, 2015 ▸) and *OLEX2* (Dolomanov *et al.*, 2009 ▸).

## full crystallographic data

### 1,3-Bis(4-bromophenyl)-1H-imidazol-3-ium tetrafluoroborate Crystal data

**Table:**

C_15_H_11_Br_2_N_2_^+^·BF_4_^−^	*F*(000) = 904
*M**_r_* = 465.89	*D*_x_ = 1.986 Mg m^−^^3^
Monoclinic, *C*2/*c*	Mo *K*α radiation, λ = 0.71073 Å
*a* = 9.5407 (2) Å	Cell parameters from 5032 reflections
*b* = 9.8272 (2) Å	θ = 2.5–28.2°
*c* = 17.0451 (3) Å	µ = 5.25 mm^−^^1^
β = 102.878 (1)°	*T* = 100 K
*V* = 1557.92 (5) Å^3^	Block, colourless
*Z* = 4	0.37 × 0.28 × 0.21 mm

### 1,3-Bis(4-bromophenyl)-1H-imidazol-3-ium tetrafluoroborate Data collection

**Table:**

Bruker SMART APEXII area detector diffractometer	1922 independent reflections
Radiation source: microfocus sealed X-ray tube, Incoatec Iµs	1748 reflections with *I* > 2σ(*I*)
Mirror optics monochromator	*R*_int_ = 0.023
Detector resolution: 7.9 pixels mm^-1^	θ_max_ = 28.3°, θ_min_ = 2.5°
ω and φ scans	*h* = −12→12
Absorption correction: multi-scan (SADABS; Krause *et al.*, 2015)	*k* = −12→13
*T*_min_ = 0.618, *T*_max_ = 0.746	*l* = −22→22
9293 measured reflections	

### 1,3-Bis(4-bromophenyl)-1H-imidazol-3-ium tetrafluoroborate Refinement

**Table:**

Refinement on *F*^2^	0 restraints
Least-squares matrix: full	Hydrogen site location: inferred from neighbouring sites
*R*[*F*^2^ > 2σ(*F*^2^)] = 0.017	H-atom parameters constrained
*wR*(*F*^2^) = 0.038	*w* = 1/[σ^2^(*F*_o_^2^) + (0.0133*P*)^2^ + 2.086*P*] where *P* = (*F*_o_^2^ + 2*F*_c_^2^)/3
*S* = 1.06	(Δ/σ)_max_ = 0.002
1922 reflections	Δρ_max_ = 0.37 e Å^−^^3^
110 parameters	Δρ_min_ = −0.29 e Å^−^^3^

### 1,3-Bis(4-bromophenyl)-1H-imidazol-3-ium tetrafluoroborate Special details

**Table:**

Geometry. All esds (except the esd in the dihedral angle between two l.s. planes) are estimated using the full covariance matrix. The cell esds are taken into account individually in the estimation of esds in distances, angles and torsion angles; correlations between esds in cell parameters are only used when they are defined by crystal symmetry. An approximate (isotropic) treatment of cell esds is used for estimating esds involving l.s. planes.

### 1,3-Bis(4-bromophenyl)-1H-imidazol-3-ium tetrafluoroborate Fractional atomic coordinates and isotropic or equivalent isotropic displacement parameters (Å^2^)

**Table:**

	*x*	*y*	*z*	*U*_iso_*/*U*_eq_	
Br1	0.79285 (2)	0.44748 (2)	0.42461 (2)	0.01694 (5)	
F1	0.60495 (11)	1.28959 (10)	0.79615 (6)	0.0298 (2)	
F2	0.43907 (13)	1.12447 (11)	0.79918 (7)	0.0379 (3)	
N1	0.54845 (13)	0.66867 (12)	0.69843 (7)	0.0130 (2)	
C1	0.71272 (15)	0.51725 (16)	0.50861 (9)	0.0153 (3)	
C2	0.63143 (15)	0.63529 (15)	0.49678 (9)	0.0150 (3)	
H2	0.613229	0.680767	0.446339	0.018*	
C3	0.57701 (15)	0.68594 (15)	0.56002 (9)	0.0145 (3)	
H3	0.520537	0.766483	0.553292	0.017*	
C4	0.60593 (15)	0.61777 (15)	0.63310 (9)	0.0139 (3)	
C5	0.500000	0.5896 (2)	0.750000	0.0140 (4)	
H5	0.500002	0.492896	0.750001	0.017*	
C6	0.53051 (16)	0.80363 (15)	0.71783 (9)	0.0161 (3)	
H6	0.556228	0.881331	0.690995	0.019*	
C7	0.73926 (16)	0.44820 (16)	0.58138 (9)	0.0175 (3)	
H7	0.793608	0.366355	0.587808	0.021*	
C8	0.68604 (16)	0.49934 (16)	0.64455 (9)	0.0165 (3)	
H8	0.704252	0.453812	0.694975	0.020*	
B1	0.500000	1.2063 (3)	0.750000	0.0185 (5)	

### 1,3-Bis(4-bromophenyl)-1H-imidazol-3-ium tetrafluoroborate Atomic displacement parameters (Å^2^)

**Table:**

	*U*^11^	*U*^22^	*U*^33^	*U*^12^	*U*^13^	*U*^23^
Br1	0.01930 (8)	0.01754 (8)	0.01556 (8)	−0.00009 (6)	0.00730 (5)	−0.00169 (6)
F1	0.0342 (6)	0.0190 (5)	0.0305 (5)	−0.0001 (4)	−0.0052 (5)	−0.0008 (4)
F2	0.0569 (7)	0.0275 (6)	0.0381 (6)	−0.0040 (5)	0.0292 (6)	0.0094 (5)
N1	0.0148 (6)	0.0119 (6)	0.0123 (6)	−0.0011 (5)	0.0028 (5)	−0.0002 (5)
C1	0.0145 (7)	0.0179 (7)	0.0140 (7)	−0.0039 (6)	0.0045 (5)	−0.0027 (6)
C2	0.0148 (7)	0.0161 (7)	0.0138 (7)	−0.0035 (6)	0.0027 (5)	0.0014 (6)
C3	0.0142 (7)	0.0127 (7)	0.0162 (7)	−0.0015 (5)	0.0024 (6)	0.0008 (5)
C4	0.0137 (6)	0.0157 (7)	0.0129 (6)	−0.0027 (6)	0.0039 (5)	−0.0024 (5)
C5	0.0167 (10)	0.0115 (9)	0.0138 (9)	0.000	0.0031 (8)	0.000
C6	0.0202 (7)	0.0110 (7)	0.0163 (7)	−0.0005 (6)	0.0025 (6)	−0.0002 (5)
C7	0.0174 (7)	0.0166 (7)	0.0184 (7)	0.0024 (6)	0.0038 (6)	0.0002 (6)
C8	0.0185 (7)	0.0167 (7)	0.0138 (7)	0.0006 (6)	0.0030 (6)	0.0023 (6)
B1	0.0280 (13)	0.0122 (11)	0.0173 (11)	0.000	0.0096 (10)	0.000

### 1,3-Bis(4-bromophenyl)-1H-imidazol-3-ium tetrafluoroborate Geometric parameters (Å, º)

**Table:**

Br1—C1	1.8953 (14)	C3—H3	0.9500
F1—B1	1.3930 (18)	C3—C4	1.387 (2)
F2—B1	1.3799 (18)	C4—C8	1.382 (2)
N1—C4	1.4364 (18)	C5—H5	0.9500
N1—C5	1.3311 (17)	C6—C6^i^	1.351 (3)
N1—C6	1.3867 (19)	C6—H6	0.9500
C1—C2	1.385 (2)	C7—H7	0.9500
C1—C7	1.387 (2)	C7—C8	1.383 (2)
C2—H2	0.9500	C8—H8	0.9500
C2—C3	1.389 (2)		
			
C5—N1—C4	123.87 (13)	N1^i^—C5—H5	125.7
C5—N1—C6	108.76 (13)	N1—C6—H6	126.5
C6—N1—C4	127.35 (12)	C6^i^—C6—N1	106.97 (8)
C2—C1—Br1	120.01 (11)	C6^i^—C6—H6	126.5
C2—C1—C7	121.65 (14)	C1—C7—H7	120.2
C7—C1—Br1	118.34 (12)	C8—C7—C1	119.54 (14)
C1—C2—H2	120.6	C8—C7—H7	120.2
C1—C2—C3	118.77 (13)	C4—C8—C7	118.90 (14)
C3—C2—H2	120.6	C4—C8—H8	120.5
C2—C3—H3	120.3	C7—C8—H8	120.5
C4—C3—C2	119.35 (14)	F1^i^—B1—F1	108.01 (18)
C4—C3—H3	120.3	F2—B1—F1	110.28 (7)
C3—C4—N1	119.62 (13)	F2^i^—B1—F1^i^	110.28 (7)
C8—C4—N1	118.58 (13)	F2^i^—B1—F1	109.77 (7)
C8—C4—C3	121.78 (14)	F2—B1—F1^i^	109.77 (7)
N1^i^—C5—N1	108.53 (18)	F2^i^—B1—F2	108.73 (19)
N1—C5—H5	125.7		
			
Br1—C1—C2—C3	−178.42 (11)	C4—N1—C5—N1^i^	−178.58 (15)
Br1—C1—C7—C8	177.89 (12)	C4—N1—C6—C6^i^	178.37 (15)
N1—C4—C8—C7	178.69 (13)	C5—N1—C4—C3	142.28 (12)
C1—C2—C3—C4	0.3 (2)	C5—N1—C4—C8	−36.09 (19)
C1—C7—C8—C4	0.7 (2)	C5—N1—C6—C6^i^	−0.25 (19)
C2—C1—C7—C8	−1.3 (2)	C6—N1—C4—C3	−36.1 (2)
C2—C3—C4—N1	−179.22 (13)	C6—N1—C4—C8	145.49 (15)
C2—C3—C4—C8	−0.9 (2)	C6—N1—C5—N1^i^	0.10 (7)
C3—C4—C8—C7	0.4 (2)	C7—C1—C2—C3	0.8 (2)

Symmetry code: (i) −*x*+1, *y*, −*z*+3/2.

### 1,3-Bis(4-bromophenyl)-1H-imidazol-3-ium tetrafluoroborate Hydrogen-bond geometry (Å, º)

**Table:**

*D*—H···*A*	*D*—H	H···*A*	*D*···*A*	*D*—H···*A*
C5—H5···F1^ii^	0.95	2.29	3.156 (2)	151
C6—H6···F2^i^	0.95	2.39	3.1854 (19)	140

Symmetry codes: (i) −*x*+1, *y*, −*z*+3/2; (ii) *x*, *y*−1, *z*.
